# Disentangling mechanisms behind the pleiotropic effects of proximal 16p11.2 BP4-5 CNVs

**DOI:** 10.1016/j.ajhg.2024.08.014

**Published:** 2024-09-26

**Authors:** Chiara Auwerx, Samuel Moix, Zoltán Kutalik, Alexandre Reymond

**Affiliations:** 1 Center for Integrative Genomics, University of Lausanne, Lausanne, Switzerland; 2 Department of Computational Biology, University of Lausanne, Lausanne, Switzerland; 3 Swiss Institute of Bioinformatics, Lausanne, Switzerland; 4 University Center for Primary Care and Public Health, Lausanne, Switzerland

**Keywords:** structural variant, genomic disorder, phenome-wide association study, multi-system disorder, pleiotropy, mediation, obesity, educational attainment, Mendelian randomization, dosage sensitivity

## Abstract

Whereas 16p11.2 BP4-5 copy-number variants (CNVs) represent one of the most pleiotropic etiologies of genomic syndromes in both clinical and population cohorts, the mechanisms leading to such pleiotropy remain understudied. Identifying 73 deletion and 89 duplication carrier individuals among unrelated White British UK Biobank participants, we performed a phenome-wide association study (PheWAS) between the region’s copy number and 117 complex traits and diseases, mimicking four dosage models. Forty-six phenotypes (39%) were affected by 16p11.2 BP4-5 CNVs, with the deletion-only, mirror, U-shape, and duplication-only models being the best fit for 30, 10, 4, and 2 phenotypes, respectively, aligning with the stronger deleteriousness of the deletion. Upon individually adjusting CNV effects for either body mass index (BMI), height, or educational attainment (EA), we found that sixteen testable deletion-driven associations—primarily with cardiovascular and metabolic traits—were BMI dependent, with EA playing a more subtle role and no association depending on height. Bidirectional Mendelian randomization supported that 13 out of these 16 associations were secondary consequences of the CNV’s impact on BMI. For the 23 traits that remained significantly associated upon individual adjustment for mediators, matched-control analyses found that 10 phenotypes, including musculoskeletal traits, liver enzymes, fluid intelligence, platelet count, and pneumonia and acute kidney injury risk, remained associated under strict Bonferroni correction, with 10 additional nominally significant associations. These results paint a complex picture of 16p11.2 BP4-5’s pleiotropic pattern that involves direct effects on multiple physiological systems and indirect co-morbidities consequential to the CNV’s impact on BMI and EA, acting through trait-specific dosage mechanisms.

## Graphical abstract

**Figure:**
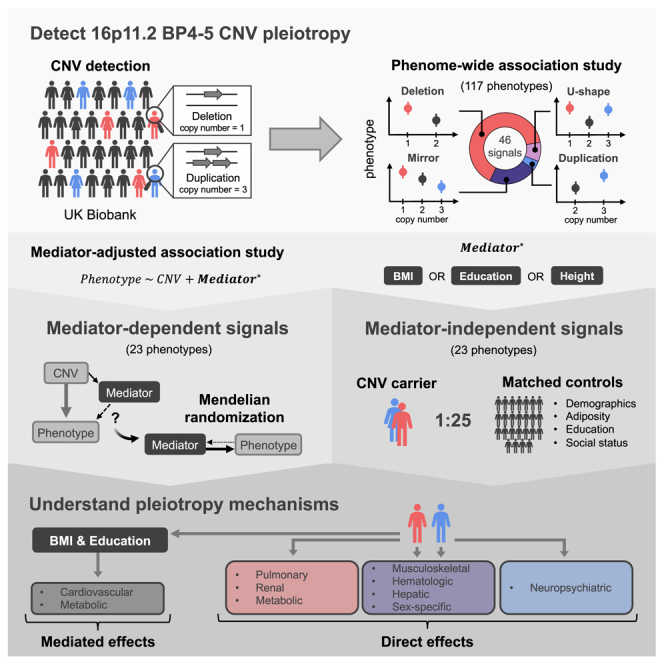

## Teaser

In the UK Biobank, 16p11.2 BP4-5 copy-number variants (CNVs) impact over 45 phenotypes across various physiological systems beyond neuropsychiatric traits. Associations are governed by distinct dosage mechanisms. Some associations are secondary to the CNV’s impact on adiposity and education level, while others are driven by independent mechanisms.

## Introduction

Genomic disorders are caused by recurrent genomic rearrangements that lead to the gain (duplication) or loss (deletion) of large, multi-kilobase pair (kb) DNA fragments. The proximal 16p11.2 rearrangement spans a region of ∼600 kb between recurrent breakpoints (BP) 4 and 5 and includes 27 unique protein-coding genes. Copy-number variants (CNVs) of the region represent one of the most common genomic disorders, with population prevalence estimates of 1 in 3,100 and 1 in 2,800 for the deletion (MIM: 611913) and duplication (MIM: 614671), respectively.^1^ Prevalence in clinical cohorts is about 8-fold higher,^1^ with a particularly strong enrichment in individuals ascertained for intellectual disability and developmental delay^2^^,^^3^^,^^4^ or autism spectrum disorder,^5^^,^^6^^,^^7^^,^^8^ the first phenotypes associated with the CNV. Other hallmark features include a negative dosage effect on body mass index (BMI)^9^^,^^10^^,^^11^ and head circumference,^12^^,^^13^ a predisposition for seizure disorders,^3^^,^^4^^,^^12^^,^^14^ and a duplication-specific increased susceptibility to schizophrenia and other psychiatric conditions.^13^^,^^15^^,^^16^^,^^17^^,^^18^ The recent establishment of large biobanks coupling genetic information to phenotypic data such as physical measurements, blood biomarkers, and electronic health records, has allowed the investigation of the phenotypic expression of 16p11.2 BP4-5 rearrangements in individuals that are typically older and less severely affected than those recruited in pediatric clinical cohorts.^19^^,^^20^^,^^21^^,^^22^^,^^23^^,^^24^^,^^25^^,^^26^^,^^27^^,^^28^^,^^29^^,^^30^ Results of these studies often converge onto similar pathophysiological processes than those highlighted by clinical studies but also report associations with biomarkers and common diseases that are typically overlooked or not assessed in clinical cohorts.^1^

**Figure 1:**
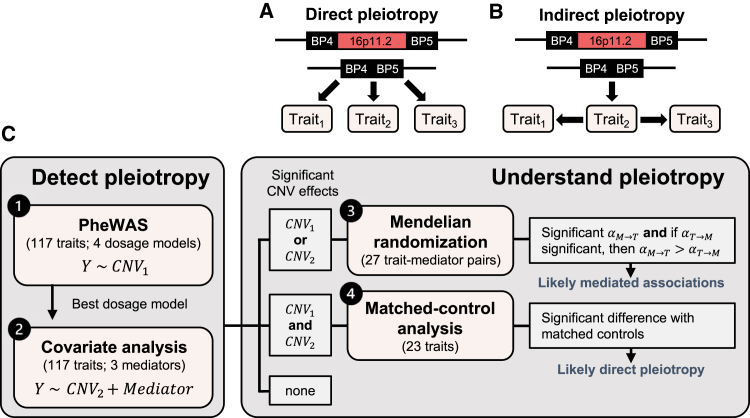
Study workflow (A) Direct (horizontal) pleiotropy: The CNV causally affects multiple traits through independent mechanisms. (B) Indirect (vertical) pleiotropy: The CNV causally impacts trait_2_, which in turn causally affects trait_1_ and trait_3_. The impact of the deletion on trait_1_ and trait_3_ is thus indirect and mediated by a shared mechanism, i.e., trait_2_. For illustration, the 16p11.2 BP4-5 deletion is shown but the same concept applies to the duplication. (C) Overview of the study. The first two analyses aim at detecting and characterizing the pleiotropy of 16p11.2 BP4-5 CNVs through four distinct dosage models that estimate the effect of the CNV on the trait (*Y*) either without (analysis 1; *CNV*_1_) or with (analysis 2; *CNV*_2_) adjustment for one of three covariates (*mediator*) that could potentially mediate the CNV-phenotype association. The second part of the study aims at understanding the mechanisms through which pleiotropy arises. Bidirectional Mendelian randomization was used to investigate the causal relationship between trait-mediator pairs for which the significance of the CNV effect on the trait was affected by adjustment for the mediator (analysis 3). Support for mediation was claimed when the forward MR effect of the mediator on the trait ($\alpha_{M\to T}$) was significant, while the reverse effect of the trait on the mediator ($\alpha_{T\to M}$) was not significant or of smaller magnitude than the forward effect. For traits that showed a significant association with the CNV regardless of covariate adjustment, matched-control analysis, which allows simultaneous adjustment for all possible mediators, was performed (analysis 4). If the effect remains significant, the association likely reflects genuine horizontal pleiotropy. BP, break point; PheWAS, phenome-wide association study.

If the pleiotropic nature of 16p11.2 BP4-5 rearrangements is now well established, the mechanisms through which CNVs in the region affect such diversity of traits remain poorly studied. Under a model of direct (or horizontal) pleiotropy, the CNV causally impacts associated phenotypes through independent mechanisms (Figure 1A). Conversely, indirect (or vertical) pleiotropy implies that the CNV causally impacts a mediatory trait, which in turn impacts other traits that will appear to be linked with the CNV in association studies (Figure 1B). These models are not mutually exclusive, and a fraction of the associations might result from direct effects while others might be secondary consequences. Resolving mechanisms of pleiotropy is particularly relevant given the BMI-modulating role of 16p11.2 BP4-5 CNVs.^9^^,^^10^^,^^11^^,^^19^^,^^20^^,^^23^^,^^24^ Indeed, BMI represents a strong risk factor for other diseases and knowledge about which associations are consequential to altered BMI could therefore inform epidemiology of associated comorbidities and clinical practice. To address this knowledge gap, we draw on our two recent UK Biobank (UKBB) studies^20^^,^^21^ to perform an in-depth analysis of the impact of 16p11.2 BP4-5 rearrangements on 117 complex traits and common diseases.^20^^,^^21^ The aims of the current study are to determine the most likely dosage mechanism for different associated traits and estimate the fraction and nature of associations that are mediated by primary changes in anthropometric measurements and cognitive ability (Figure 1C).

## Material and methods

### Study material

#### Software versions

Statistical analyses and graphs were generated with R v.4.3.1. The Mendelian randomization analysis pipeline was implemented in R v.4.2.1 and uses the TwoSampleMR R package v.0.5.7^31^ and PLINK v.1.9.^32^

#### Cohort description and sample selection

Analyses were carried out in the UKBB, a volunteer-based UK population cohort of about half a million individuals (54% females) aged 40–69 years at recruitment.^33^ UKBB has approval from the North West Multi-centre Research Ethics Committee (MREC) as a Research Tissue Bank (RTB) and all participants signed a broad informed consent form. UKBB data are available for registered users and were accessed through the application number 16389. Available data include microarray genotype data acquired in GRCh37/hg19 from two similar arrays, as well as rich phenotypic data, including anthropometric measurements, vital signs, blood biomarker levels, life history and lifestyle questionnaire data, hospital-based International Classification of Diseases, 10^th^ Revision (ICD-10) codes (up to September 2021), and self-reported conditions. Analyses conducted in this study focus on 331,522 unrelated individuals from the White British UKBB subset (54% females) that were filtered to exclude samples with abnormal CNV profiles and/or a report of blood malignancy. Filtering criteria to obtain this subset are described elsewhere.^20^^,^^21^

#### CNV carrier individual identification

CNV calls from a previous study were used.^20^ Briefly, CNV calling was done based on the UKBB microarray data using standard PennCNV v.1.0.5 settings.^34^ Each call was attributed a quality score ranging from −1 (likely deletion) to 1 (likely duplication) reflecting the probability for the CNV to be a consensus call across three algorithms and thus a true positive.^35^ 16p11.2 BP4-5 deletion and duplication carrier individuals were identified as carrying a high-confidence CNV call (quality score < −0.5 for deletions; quality score > 0.5 for duplications) on chromosome 16 with start and end site within 29.40–29.80 Mb and 30.05–30.40 Mb, respectively. Individuals with a low-quality 16p11.2 BP4-5 CNV were excluded (set as N/A) from all analyses. CNV genotype vectors were then encoded to allow the fitting of regression models according to four dosage mechanisms (Table 1).

**Table 1:** Encoding of CNV carrier status for different dosage models

	**Deletion (copy number = 1)**	**Copy-neutral (copy number = 2)**	**Duplication (copy number = 3)**
Mirror	−1	0	1
U-shape	1	0	1
Duplication-only	N/A	0	1
Deletion-only	1	0	N/A

Numerical encoding of CNV genotypes for high-confidence deletion carrier individuals, copy-neutral individuals, and high-confidence duplication carrier individuals according to four dosage mechanisms of action. Individuals with a low-quality score CNV call are set as missing (N/A).

#### Phenotype selection

We analyzed the same 117 phenotypes as defined in previous studies.^20^^,^^21^ This includes 57 quantitative traits^20^ that were inverse normal transformed before being corrected for sex (except for sex-specific traits), age (UKBB field identifier #21003), age^2^, genotyping batch, and principal components 1 to 40. We further include 60 common diseases based on ICD-10 clinical diagnoses using a case-control definition procedure that excludes from controls individuals with a condition related to the one under investigation.^21^

#### Mediator selection

We tested the role of three factors that could potentially mediate associations between 16p11.2 BP4-5 CNVs and the assessed phenotypes.

(1) Body mass index (BMI): average over available instances of BMI (UKBB field identifier #21001).
(2) Educational attainment (EA): age at which full-time education was completed (UKBB field identifier #845). Values matching “prefer not to answer,” “never went to school,” and “do not know” were set as missing, and average over available instances was calculated. Individuals for which average age at which full-time education was completed was below 14 years or over 19 years were set to 14 years and 19 years, respectively. Individuals reporting a “college or university degree” in their qualifications (UKBB field identifier #6138) were set to 19 years.
(3) Height: average over available instances of standing height (UKBB field identifier #50).

#### GWAS summary statistics

Mendelian randomization (MR) studies rely on publicly available genome-wide association study (GWAS) summary statistics computed for individuals (males and females combined) of European ancestry. For mediators, summary statistics from Pan-UK Biobank (manifest updated 01/03/2023; https://pan.ukbb.broadinstitute.org/)^36^ were used for BMI and height, while a large meta-analysis was used for EA (excluding 23andMe data; http://www.thessgac.org).^37^ For other phenotypes, summary statistics from the Neale group were used (released 07/2018; http://www.nealelab.is/uk-biobank). The latter were favored over those of large disease-specific consortia as summary statistics for binary traits were calculated through linear regression, allowing comparison of forward and reverse effects. For diseases, we used the closest possible match to our phenotype definition, i.e., phenotype code E10 for T1D (type 1 diabetes); G47 for sleep (sleep apnea); I10 for HTN_essential (essential hypertension); I35 for valves (cardiac valve disorders); I44 for conduction (cardiac conduction disorders); J45 for asthma; M19 for OA (arthrosis); N18 for CKD (chronic kidney disease); and 20002_1473 for lipid (lipidemias & lipoprotein disorders). We expect sample overlap between mediator and trait summary statistics to only minimally bias MR estimates.^38^ Summary statistics for autosomal chromosomes were harmonized to the UK10K reference panel.^39^ After excluding palindromic single-nucleotide polymorphisms (SNPs) and adjusting strand-flipped SNPs, effect sizes were standardized to represent the square root of the explained variance.

### 16p11.2 BP4-5 association studies

#### Phenome-wide association study

For the phenome-wide association study (PheWAS), regression analysis was performed to estimate the effect of the CNV genotype—encoded according to either of the four models in Table 1—and the 117 selected phenotypes. For quantitative traits, linear regressions (lm() in R) were used and 95% confidence intervals (CI) were calculated as the effect size ±1.96 × standard error (SE). For binary traits, Firth’s bias-reduced penalized-likelihood logistic regression was used (logistf(plconf = 2, maxit = 100, maxstep = 10) from the logistf package v.1.26.0 in R) to account for the fact that both CNV carrier individuals and disease cases are rare. The same function also produces estimates for the 95% CIs. As disease diagnoses were defined as binary variables and could not be adjusted beforehand, sex (except for sex-specific traits), age, genotyping array, and principal components 1 to 40 were included in the model as covariates. For each trait, the dosage model yielding the lowest *p* value for the CNV effect was retained and effects were defined as strictly significant under Bonferroni correction criteria (*p* ≤ 0.05/117 = 4.3 × 10^−^^4^). As a sensitivity analysis, we further report the number of associations surviving a stricter Bonferroni correction accounting for the four tested models (*p* ≤ 0.05/(4 × 117) = 1.1 × 10^−^^4^), even though it should be noted that this results in an overly strict correction as the four models are not independent.

#### Covariate analysis

For all 351 pairs between the 117 phenotypes (including those involving phenotypes that did not significantly associate with the CNV status in our original PheWAS) and 3 putative mediators (phenotype selection and mediator selection), we estimated the Pearson correlation (cor(use = “pairwise.complete.obs”) in R), as well as the effect of the mediator on the phenotype in a linear/Firth regression model without covariates, as previously described (phenome-wide association study). Pairs with a Pearson correlation coefficient <0.7 and an effect of the mediator on the trait meeting *p* ≤ 0.05/351 = 1.4 × 10^−^^4^ were retained. For them, the effect of the CNV carrier status encoded according to the best PheWAS model was estimated through regression analysis (phenome-wide association study), adding the mediator as an additional covariate. Adjusted effects were defined as strictly significant when meeting Bonferroni correction criteria (*p* ≤ 0.05/117 = 4.3 × 10^−^^4^). The difference in correlation between BMI-dependent and BMI-independent traits with BMI was assessed with a two-sided test.

#### Mendelian randomization

SNP-GWAS summary statistics were used to conduct bidirectional MR according to a previously published pipeline^40^^,^^41^ for 27 mediator-trait pairs for which the CNV-trait association either gained or lost significance upon adjusting for that mediator. Importantly, CNVs are not used as instrumental variables in the MR analyses. Concretely, the forward effect of the mediator (exposure) on the trait (outcome) and the reverse effect of the trait (exposure) on the mediator (outcome) were estimated. Harmonized SNPs significantly (*p* < 5 × 10^−^^8^) associating with the exposure were clumped with PLINK v.1.9 (--clump --clump-p1 0.0001 --clump-p2 0.01 --clump-kb 250 --clump-r2 0.01) and retained as instrumental variables. Instrumental variables mapping to the extended HLA region (chr6:25,000,000–37,000,000 [GRCh37/hg19]) were excluded, as well as those with a difference in allele frequency (≥0.05) between the outcome and exposure summary statistic. Steiger filtering was applied (Z ≤ −1.96) to ensure that the effect of the selected variants on the exposure was stronger than their effect on the outcome. Bidirectional inverse-variance weighted (IVW) MR analyses were carried out with the TwoSampleMR R package when at least two instrumental variables were available. IVW MR effects were called significant under Bonferroni correction, when *p* ≤ 0.05/54 = 9.3 × 10^−^^4^, to account for the 27 bidirectional tests performed. As a sensitivity analysis, we assessed three additional MR methods implemented in the TwoSampleMR R package, i.e., simple mode, weighted median, and weighted mode MR.

#### Matched-control analysis

For each CNV carrier individual, we identified all copy-neutral unrelated individuals from the White British subset of UKBB participants that were matching based on sex (identical), age (±2.5 years), BMI (±2.5 kg/m^2^), Townsend deprivation index at recruitment (UKBB field identifier #22189; ±2), and average household income before tax (UKBB field identifier #738) averaged over instances (identical category), and EA (±1 year). Fifty-eight deletion and sixty-one duplication carrier individuals had no missing data and qualified for the matching procedure. The number of identified matching control subjects per carrier individual ranged from 1 to 918 and 12 to 1,590 for deletion and duplication carrier individuals, respectively, with 49 deletion and 60 duplication carrier individuals having at least 25 matching control subjects. When more than 25 matched control subjects were available, the ones used for the analysis were selected randomly (sample_n() in R), without replacement. For quantitative traits, we compared mean phenotypic values between deletion and duplication carrier individuals and the respective control groups through a two-sided t test. For binary traits, disease prevalence was compared between the same groups based on a two-sided Fisher test. Prevalence SE was calculated as

$$SE=\sqrt{\frac{q*\left(1-q\right)}{n}}$$

where *q* is the disease prevalence in a given group and *n* is its sample size. Sample sizes vary between phenotypes due to missing data. We define significant associations based on a Bonferroni correction that accounts for the 23 traits of interest in this analysis (*p* ≤ 0.05/23 = 2.2 × 10^−^^3^), i.e., phenotypes that remained associated with the CNV under strict Bonferroni correction when adjusting for BMI, height, or EA individually. We report all nominally significant (*p* < 0.05) associations in the figures. As an additional quality control assessing the consequences of losing samples for the matched-control analysis, we used the same statistical framework to compare mean phenotypic value and disease prevalence between deletion and duplication carrier individuals that were included in the matched-control analysis versus those that were not due to missing data or lack of sufficient control subjects.

## Results

### Phenome-wide association study

**Figure 2:**
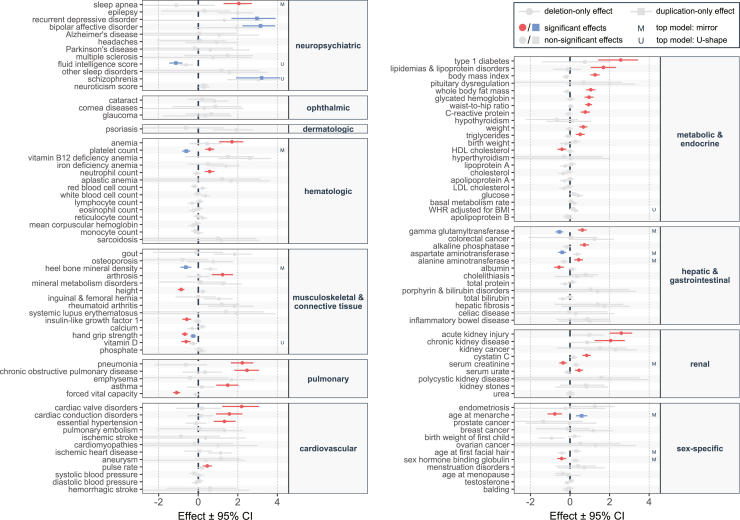
16p11.2 BP4-5 phenome-wide association study Effect sizes (beta; x axis) with 95% confidence interval (CI) of the 16p11.2 BP4-5 deletion (circle) and duplication (square) on 117 complex traits and diseases, ordered by physiological system (y axis). Effect sizes are in standard deviation units of the outcome (quantitative traits) or logarithms of the odds ratio of a logistic regression (disease traits). Deletion- and duplication-only effects that are significant under Bonferroni correction (*p* ≤ 0.05/117 = 4.3 × 10^−^^4^) are in blue and red, respectively, while non-significant effects are in gray. If the most significant model was the mirror or U-shape model, it is denoted with an “M” or “U,” respectively (right). BMI, body mass index; HDL, high-density lipoprotein; LDL, low-density lipoprotein; WHR, waist-to-hip ratio.

Using previously published high-confidence CNV calls for 331,522 unrelated, White British UKBB participants,^20^^,^^21^ we identified 73 and 89 individuals with a 16p11.2 BP4-5 (start: 29.40–29.80 Mb; end: 30.05–30.40 Mb) deletion and duplication, respectively. CNV genotypes were encoded to allow testing of four dosage mechanisms, namely a mirror model assessing the additive impact of each additional copy, a U-shape model testing the same-direction impact of any deviation from the copy-neutral state, and duplication- and deletion-only models that assess the separate impact of duplications and deletions, respectively (Table 1). Next, we evaluated the association between an individual’s CNV carrier status and 117 phenotypes—that comprise 57 quantitative variables including anthropometric measurements, vital signs, biomarker levels, life history events, and 60 common diseases—while correcting for sex, age, genotyping array, and population stratification (Figure 2; Table S1).

Overall, 46 (39%) traits, including 16 diseases, were associated with the CNV carrier status under at least one association model (Bonferroni correction: *p* ≤ 0.05/117 = 4.3 × 10^−^^4^; Tables 2 and 3), with an additional 32 (27%) showing a trend for association (nominal significance: *p* ≤ 0.05). Importantly, only five associations—with high-density lipoprotein (HDL) cholesterol levels, waist-to-hip ratio (WHR) adjusted for BMI, pulse rate, type 1 diabetes, and recurrent depressive disorder—were lost when further accounting for the four tested association models, corroborating the statistical robustness of our findings. Among the 46 Bonferroni-corrected associations, 10 and 38 traits showed a significant association through the duplication-only and deletion-only models, respectively, indicating a stronger propensity for pleiotropy and deleteriousness of the deletion, compared to the duplication. Exceptions are recurrent depressive disorder and bipolar affective disorder, the only two traits for which the duplication-only model yielded the most significant result. This is in line with the duplication representing a strong susceptibility factor for psychiatric conditions.^13^^,^^15^^,^^16^^,^^17^^,^^18^ Similarly, the risk for schizophrenia was strongly increased by the duplication, even if our analysis finds that the relation is better described by a U-shape model wherein the deletion also tends to increase schizophrenia risk. Surprisingly, the CNV did not associate with neuroticism score, despite the high genetic correlation between neuroticism and psychiatric conditions.^42^ Three other traits, namely fluid intelligence, vitamin D levels, and WHR adjusted for BMI, were also most significantly associated through a U-shape effect, while hand grip strength was decreased in both deletion and duplication carrier individuals, but more strongly in the former. Conversely, ten traits were most significantly associated through a mirror model, including multiple hepatic biomarkers, platelet count, and traits related to sexual characteristics such as puberty timing and sex hormone binding globulin (SHBG) levels. Finally, the deletion-only model was the most significant fit for 30 phenotypes, including mostly pulmonary, cardiovascular, metabolic, and renal traits.

**Table 2:** Quantitative traits significantly associated with 16p11.2 BP4-5 CNVs

**CNV status**		**Controls**	**Deletion carriers**		**Duplication carriers**	
Phenotype	**Unit**	**Mean**	***n***	**Mean ± SE**	***n***	**Mean ± SE**
Fluid intelligence score	points	6.24	26	5.15 ± 0.39	32	4.05 ± 0.37
Platelet count	10^9^ cells/L	252.8	70	286.4 ± 7.5	85	219.4 ± 5.7
Neutrophil count	10^9^ cells/L	4.25	70	5.00 ± 0.15	85	4.14 ± 0.15
Heel bone mineral density	g/cm^2^	0.54	29	0.66 ± 0.04	47	0.46 ± 0.01
Height	cm	168.8	73	163.3 ± 1.0	89	171.1 ± 1.1
Insulin-like growth factor 1	nmol/L	21.4	71	18.9 ± 0.7	88	21.4 ± 0.6
Hand grip strength	kg	30.7	73	26.7 ± 1.0	89	28.6 ± 1.2
Vitamin D	nmol/L	49.8	68	36.0 ± 2.1	86	44.7 ± 2.2
Forced vital capacity	L	3.63	64	2.94 ± 0.11	72	3.64 ± 0.12
Pulse rate	bpm	69.3	67	74.3 ± 1.5	81	71.3 ± 1.3
Body mass index	kg/m^2^	27.4	72	35.0 ± 0.9	89	26.3 ± 0.4
Whole body fat mass	kg	24.9	67	35.5 ± 1.6	85	23.2 ± 0.9
Glycated hemoglobin	mmol/mol	36.0	70	43.3 ± 1.8	85	35.7 ± 0.5
Waist-to-hip ratio	–	0.87	72	0.97 ± 0.01	89	0.87 ± 0.01
C-reactive protein	m/L	2.57	71	5.50 ± 0.76	88	2.03 ± 0.23
Weight	kg	78.3	72	93.3 ± 2.7	89	77.1 ± 1.4
Triglycerides	mmol/L	1.75	71	2.30 ± 0.14	87	1.60 ± 0.09
HDL cholesterol	mmol/L	1.46	61	1.26 ± 0.04	79	1.45 ± 0.04
WHR adjusted for BMI	–	0.00	71	0.03 ± 0.01	89	0.01 ± 0.01
Gamma-glutamyltransferase	U/L	37.3	71	64.1 ± 11.3	87	27.7 ± 2.8
Alkaline phosphatase	U/L	83.6	71	99.8 ± 3.4	88	79.8 ± 2.6
Aspartate aminotransferase	U/L	26.2	70	35.8 ± 6.7	87	23.4 ± 0.7
Alanine aminotransferase	U/L	23.5	71	31.2 ± 2.1	88	20.8 ± 1.3
Albumin	g/L	45.3	61	44.0 ± 0.4	79	45.6 ± 0.3
Cystatin C	mg/L	0.91	71	1.04 ± 0.02	88	0.93 ± 0.02
Serum creatinine	μmol/L	72.4	71	68.8 ± 1.6	87	76.9 ± 2.0
Serum urate	μmol/L	309.4	70	355.2 ± 8.1	88	298.8 ± 9.1
Age at menarche	years	12.9	27	11.7 ± 0.3	45	14.0 ± 0.4
Age at first facial hair (group 1–3)	–	2.06	41	1.83 ± 0.06	38	2.26 ± 0.08
Sex hormone binding globulin	nmol/L	51.9	61	38.4 ± 2.4	77	58.8 ± 3.4

Quantitative traits that are significantly (*p* ≤ 0.05/117 = 4.3 × 10^−^^4^) associated with 16p11.2 BP4-5 CNVs through at least one of the four assessed association models, following the ordering of Figure 2. The mean value of the traits in copy-neutral individuals (control subjects) is provided along with the mean value and standard error (SE) among deletion and duplication carrier individuals. The number of duplication and deletion carrier individuals with available data is specified as *n*. Values are given in the indicated unit. HDL, high-density lipoprotein; WHR, waist-to-hip ratio.

**Table 3:** Diseases significantly associated with 16p11.2 BP4-5 CNVs

**CNV status**	**Controls**	**Deletion carriers**		**Duplication carriers**	
Disease	**Prevalence**	**Case/*n***	**Prevalence ± SE**	**Case/*n***	**Prevalence ± SE**
Sleep apnea	2.1%	9/60	15.0% ± 4.6%	0/70	0.0% ± 0.0%
Recurrent depressive disorder	0.3%	0/49	0.0% ± 0.0%	3/68	4.4% ± 2.5%
Bipolar affective disorder	0.4%	1/50	2.0% ± 2.0%	6/71	8.5% ± 3.3%
Schizophrenia	0.2%	2/51	3.9% ± 2.7%	3/68	4.4% ± 2.5%
Anemia	5.5%	13/67	19.4% ± 4.8%	6/84	7.1% ± 2.8%
Arthrosis	21.1%	23/62	37.1% ± 6.1%	15/74	20.3% ± 4.7%
Pneumonia	5.9%	19/61	31.1% ± 5.9%	2/83	2.4% ± 1.7%
Chronic obstructive pulmonary disease	4.9%	17/55	30.9% ± 6.2%	4/76	5.3% ± 2.6%
Asthma	12.1%	19/55	34.5% ± 6.4%	9/69	13.0% ± 4.1%
Cardiac valve disorders	5.0%	7/33	21.2% ± 7.1%	3/54	5.6% ± 3.1%
Cardiac conduction disorders	19.5%	18/44	40.9% ± 7.4%	13/64	20.3% ± 5%
Essential hypertension	35.3%	36/62	58.1% ± 6.3%	23/74	31.1% ± 5.4%
Type 1 diabetes	1.0%	4/42	9.5% ± 4.5%	1/75	1.3% ± 1.3%
Lipidemias & lipoprotein disorders	22.0%	23/48	47.9% ± 7.2%	11/57	19.3% ± 5.2%
Acute kidney injury	4.7%	20/65	30.8% ± 5.7%	7/71	9.9% ± 3.5%
Chronic kidney disease	4.4%	9/54	16.7% ± 5.1%	6/70	8.6% ± 3.3%

Binary disease traits that are significantly (*p* ≤ 0.05/117 = 4.3 × 10^−^^4^) associated with 16p11.2 BP4-5 CNVs through at least one of the four assessed association models, following the ordering of Figure 2. Prevalence in percentage among copy-neutral individuals (control subjects) is provided along with prevalence and standard error (SE) among deletion and duplication carrier individuals. Diseased (case) and total (*n*) number of deletion and duplication carrier individuals are indicated.

**Figure 3:**
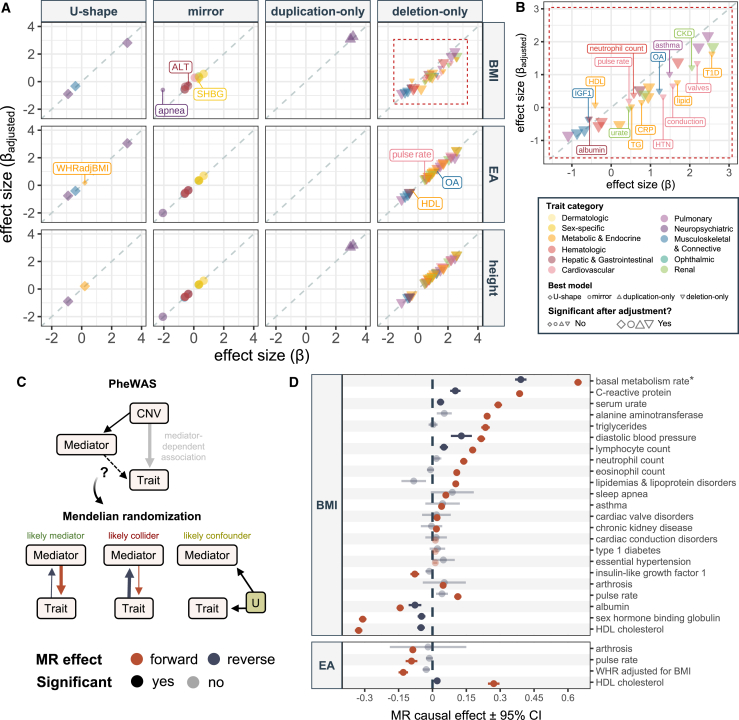
Mediation of 16p11.2 BP4-5 pleiotropy through anthropometric traits and educational attainment (A) Effects (beta) of 16p11.2 BP4-5 CNVs on traits with adjustment for potential mediators (y axis)—i.e., body mass index (BMI; top row), educational attainment (EA; middle row), or height (bottom row)—against those without adjustment (x axis), stratified according to the best (i.e., most significant) association model (shape; columns). Only associations that were significant prior to or become significant after adjustment are plotted. Traits are colored according to physiological systems. Size reflects whether the effect is Bonferroni significant (*p* ≤ 0.05/117 = 4.3 × 10^−^^4^) after adjusting for the potential mediator. Traits losing significance upon adjustment are labeled (see B for BMI-adjustment of deletion-only associations). Gray dashed diagonal represents the identity line. (B) Enlargement of the area delimited by a red dashed rectangle in (A), showing the effect of BMI adjustment for deletion-driven associations, using the same legend as in (A). (C) Schematic representation of the links between 16p11.2 BP4-5 CNVs, potential mediators, and assessed traits. Covariate-adjusted phenome-wide association studies (PheWASs) identified CNV-trait associations that are dependent on either of two tested factors (i.e., BMI or EA; thick gray arrow) in (A). This can happen through mediation, collider bias, or confounding. Bidirectional Mendelian randomization (MR) assesses the genetically determined causal effect of the putative mediator on the trait (forward effect, red arrow) and of the trait on the mediator (reverse effect; dark blue arrow), with arrows proportional to causal effect sizes. When the forward effect is larger than the reverse one, mediation is a likely scenario; when the reverse effect is larger, the putative mediator likely acts as a collider; absence of causal effects likely indicates presence of an unobserved confounder, U. Depending on the scenario, adjustment for the mediator in the regression analysis might or might not be appropriate. (D) Bidirectional forward (red) and reverse (dark blue) MR effects with 95% confidence interval (CI) of potential mediators (left y axis) on traits (right y axis) for all mediator-trait pairs that either gained or lost significance upon adjustment for the mediator. Phenotypes labeled with “^∗^” exhibit a Pearson correlation between 0.4 and 0.7 with the mediator. Non-significant effects (*p* > 0.05/54 = 9.3 × 10^−^^4^) are semi-transparent. ALT, alanine aminotransferase; apnea, sleep apnea; CKD, chronic kidney disease; conduction, cardiac conduction disorders; CRP, C-reactive protein; HDL, high-density lipoprotein; HTN, essential hypertension; IGF1, insulin-like growth factor 1; lipid, lipidemias & lipoprotein disorders; OA, arthrosis; SHBG, sex hormone binding globulin; T1D, type 1 diabetes; TG, triglycerides; urate, serum urate; valves, cardiac valve disorders; WHRadjBMI, waist-to-hip ratio (WHR) adjusted for BMI.

Having characterized the pleiotropic nature of 16p11.2 BP4-5 rearrangements, we next sought to establish whether some of these associations might be secondary to the CNV affecting core mediatory phenotypes, i.e., reflect indirect pleiotropy (Figure 1B). We focus on three traits that proxy hallmark features of the 16p11.2 BP4-5 rearrangement and have the potential to influence other associated traits: (1) BMI, which characterizes the negative correlation between dosage and adiposity^9^^,^^10^^,^^11^^,^^20^^,^^24^^,^^43^ and represents a major risk factor for many common diseases; (2) height, which is reduced in deletion carrier individuals^20^^,^^24^^,^^43^ and can influence musculoskeletal phenotypes; and (3) EA proxied by age at which an individual completed their education, which reflects the decreased cognitive function observed in both duplication and deletion carrier individuals.^3^^,^^4^^,^^16^^,^^20^^,^^30^^,^^43^^,^^44^ The latter variable offers the advantage of being available for the near totality of the UKBB cohort while strongly correlating with fluid intelligence score that is limited to about half of its participants (Pearson correlation = 0.42). For an association between CNV and phenotype to be mediated by one of these factors, the mediator needs to significantly (*p* ≤ 0.05/351 = 1.4 × 10^−^^4^) associate with the tested phenotype. Furthermore, phenotypes cannot be too correlated with the mediator (Pearson’s correlation < 0.7), as this would induce multicollinearity in the regression model that could bias effect size estimates. For all mediator-trait pairs that fulfilled these criteria (Figure S1; Table S2), we tested the impact of adjusting the CNV-trait effect for mediatory factors by including them individually in the regression model yielding the most significant CNV-trait effect (Figure 3A; Table S3).

Upon adjustment for BMI, EA, and height, 19, 4, and 0 previously Bonferroni significant (*p* ≤ 0.05/117 = 4.3 × 10^−^^4^) CNV-trait associations lost their association to the CNV, respectively. Remarkably, the deletion-only associations with basal metabolic rate, eosinophil count, and lymphocyte count, as well as the mirror association with diastolic blood pressure, became significant upon adjustment for BMI (Figure S2A). The impact of adjusting for BMI was most striking on deletion-driven associations, 16 of which were lost (Figure 3B). In line with expectations, these BMI-dependent traits tended to have a stronger correlation with BMI than those that remained significant upon adjustment for BMI (*p* = 0.218) (Figure S2B). Among the lost associations were nine metabolic and cardiovascular traits, such as levels of serum lipid and the inflammation biomarker C-reactive protein, cardiac valve and conduction disorders, and essential hypertension. These likely reflect secondary consequences of the propensity for obesity induced by the deletion. The effect of BMI on musculoskeletal, pulmonary, or renal traits is more balanced, with some associations, such as the ones with arthrosis, asthma, or urate levels and chronic kidney disease (CKD), appearing to be driven by BMI, while others, such as hand grip strength, chronic obstructive pulmonary disease, or cystatin C levels and acute kidney injury (AKI), remaining significant upon BMI adjustment. The mediating role of EA was milder, as only four associations were lost upon adjustment for either variable, namely WHR adjusted for BMI, pulse rate, HDL cholesterol level, and arthrosis risk. Surprisingly, associations with psychiatric disorders were not affected by EA, suggesting that cognition and psychiatric diseases are regulated by (at least partially) independent pathways. Finally, the observation that no associations were affected by adjusting for height confirms that the decrease in traits such as hand grip strength and forced vital capacity among deletion carrier individuals are not driven solely by their short stature.

### Mediated CNV effects

One caveat of our analysis is that it cannot distinguish whether changes in CNV-trait associations are indeed secondary effects of the mediator on the trait. At least three scenarios could result in the loss (or gain) of a CNV-trait effect upon covariate adjustment (Figure 3C). The first one is mediation, wherein the CNV affects the trait through the mediator, resulting in a dominant causal effect of the mediator on the trait. The second scenario is when the variable we adjusted for turns out to be a collider of the CNV and the trait, in which case we expect a dominant causal effect from the trait to the “mediator.” Finally, data could be explained by an unobserved confounder that affects both the adjustment variable and the trait, in which case we do not expect any causal link between trait and mediator. Of note, in the latter scenario, we further distinguish between whether the CNV has an impact on the confounder, the “mediator,” the trait, or a combination thereof. Importantly, adjusting for the mediator in the regression model is an appropriate solution to obtain meaningful direct CNV-trait effects (i.e., genuine direct pleiotropy) only in the (1) mediator scenario or (2) the confounder scenario where the CNV has a direct effect on the trait, in which case adjustment for the mediator could result in a gain of power (Figure 3C). To identify cases where mediation is a likely scenario, we turned to bidirectional IVW MR, a causal inference approach that allows to estimate the genetically determined causal effect of an exposure on an outcome (Figure 3D; Table S4). Firstly, we estimated the forward mediator-to-trait effect for all 27 mediator-trait pairs that either gained (*n* = 4; Figure S2A) or lost (*n* = 23; Figures 3A and 3B) significance upon adjustment for either putative mediator. Except for the effect of BMI on essential hypertension, type 1 diabetes, and cardiac conduction disorders, all effects were significant (*p* ≤ 0.05/54 = 9.3 × 10^−^^4^), confirming that the mediators can causally influence the involved traits. Secondly, we estimated the reverse trait-to-mediator causal effects. Nine reverse effects were significant and thus represent mediator-trait pairs at risk for collider bias. Yet, for all of them, the forward effect had a larger magnitude, making the mediator-to-trait causal path more likely. It is also worth noting that the three associations lacking a significant forward effect also lacked a significant reverse effect, possibly indicating presence of an unobserved confounder. This is particularly likely as estimates are close to null despite being well instrumented (≥50 instrumental variables). Because IVW can be prone to false positives, we replicated our analyses with three additional MR methods, i.e., simple mode, weighted median, and weighted mode MR (Figure S3; Table S4). These yielded overall consistent results, yet it is noteworthy that the effect of BMI on CKD did not reach the significance threshold through other methods, despite consistent effect size estimates. Importantly, these results did not change conclusions about the dominant effect direction. Globally, these analyses support that a large fraction (89%) of the flagged associations are likely indirect consequences of the CNV’s effect on our selected mediators.

### Direct CNV effects

**Figure 4:**
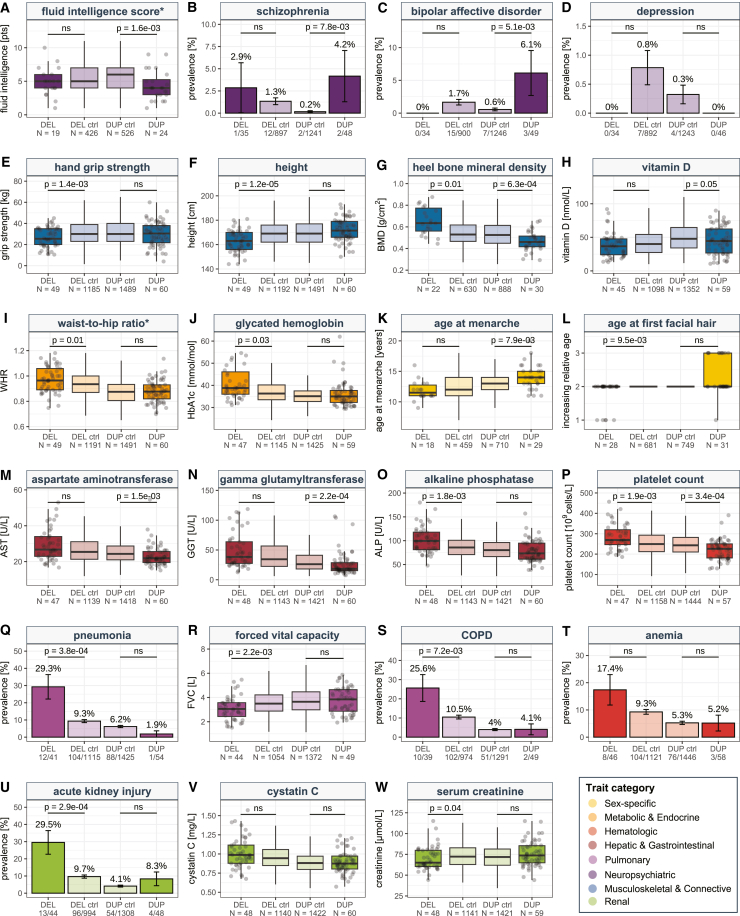
16p11.2 BP4-5 CNV carrier individuals matched-control analyses Comparison between deletion (DEL) and duplication (DUP) carrier individuals (darker shade) and their respective matched control subjects (DEL or DUP “ctrl”; lighter shade) for 23 traits that remained Bonferroni-significant (*p* ≤ 0.05/117 = 4.3 × 10^−^^4^) after individually adjusting for body mass index (BMI), educational attainment (EA), or height in Figure 3. Phenotypes labeled with “^∗^” exhibit a Pearson correlation between 0.4 and 0.7 with either BMI or EA. For quantitative traits, data are represented as boxplots without outliers: boxes show the first (Q1), second (median, thick line), and third (Q3) quartiles; lower and upper whiskers show the most extreme value within Q1 minus and Q3 plus 1.5 × the interquartile range, respectively. Data points for CNV carrier individuals are shown as gray dots. Sample size is indicated as *n*. *p* values of two-sided t tests comparing CNV carrier individuals to matched control subjects are reported. For diseases, prevalence in percentage with standard errors is depicted. Number of cases over total sample size is indicated. *p* values of two-sided Fisher tests comparing CNV carrier individuals to matched control subjects are reported. “ns” indicates *p* > 0.05. Traits are colored according to physiological systems. ALP, alkaline phosphatase; AST, aspartate aminotransferase; BMD, bone mineral density; COPD, chronic obstructive pulmonary disease; depression, recurrent depressive disorder; FVC, forced vital capacity; GGT, gamma-glutamyltransferase; HbA1c, glycated hemoglobin; WHR, waist-to-hip ratio.

Next, we focused on the 23 traits whose association with the CNV remained significant after adjusting for BMI, EA, and height. To confirm that these represent cases of genuine direct pleiotropy, we used a matched-control approach that offers the advantage of allowing adjustment for multiple mediatory variables at once but at the cost of losing some statistical power. Specifically, for each of the 58 deletion and 61 duplication carrier individuals with sufficient data to carry out the matching, we identified individuals with matched age (±2.5 years), sex (identical), BMI (±2.5 kg/m^2^), Townsend deprivation index (±2), income class (identical), and EA (±1 year) among a pool of copy-neutral, unrelated, White British UKBB participants (Figure S4). For 49 deletion and 60 duplication carrier individuals, at least 25 matched control subjects could be identified, and phenotype mean or disease prevalence between the two CNV groups and their respective control subjects were compared (Figure 4; Tables S5 and S6). Ten traits retained a strictly significant effect (*p* ≤ 0.05/23 = 2.2 × 10^−^^3^), affecting six independent physiological systems: musculoskeletal, neuropsychiatric, pulmonary/immune, renal, hepatic, and hematological. Specifically, deletion carrier individuals presented with decreased hand grip strength (*p* = 1.4 × 10^−^^3^; Figure 4E), shorter stature (*p* = 1.2 × 10^−^^5^; Figure 4F), increased alkaline phosphatase levels (*p* = 1.8 × 10^−^^3^; Figure 4O), and increased risk for pneumonia (*p* = 3.8 × 10^−^^4^; Figure 4Q) and AKI (*p* = 2.9 × 10^−^^4^; Figure 4U). Duplication carrier individuals showed reduced fluid intelligence score (*p* = 1.6 × 10^−^^3^; Figure 4A), decreased heel bone mineral density (*p* = 6.3 × 10^−^^4^; Figure 4G), and lower aspartate aminotransferase (*p* = 1.5 × 10^−^^3^; Figure 4M) and gamma-glutamyltransferase (*p* = 2.2 × 10^−^^4^; Figure 4N) levels. Noteworthy is the strong mirror effect on platelet count (Figure 4P), with higher (*p* = 1.9 × 10^−^^3^) and lower (*p* = 3.4 × 10^−^^4^) counts observed in deletion and duplication carrier individuals, respectively. Whereas for most phenotypes the other CNV type did not meet the strict significance criteria, all effects showed a trend for a mirror effect, except for fluid intelligence score and AKI, which showed a U-shape trend. Besides reinforcing its long-established consequence on cognitive function, our results assert the role of the hepatic, musculoskeletal, and pulmonary systems in the 16p11.2 BP4-5 pathophysiology through mechanisms that are independent of the CNV’s impact on anthropometric traits and education.

Finally, we performed sensitivity analyses to validate the robustness of our conclusions. As a negative control, we performed the matched-control analysis for the 23 traits that were significantly associated with 16p11.2 BP4-5 CNVs in our PheWAS but whose association was dependent on adjustment for mediators or that could not be tested in the covariate analysis due to high trait-mediator correlation (Figure S5; Tables S5 and S6). In line with these associations being secondary consequences to the effect of the CNV on factors on which the matching was performed, only two traits had a nominally significant CNV association, and none survived Bonferroni correction. This strongly contrasts with our main matched-control analysis, where only three traits lacked a nominally significant effect: recurrent depressive disorder (Figure 4D), anemia (Figure 4T), and cystatin C (Figure 4V). This absence of association could be either the result of a loss in statistical power resulting from CNV carrier subsampling or the consequence of these associations being driven by a combination of factors on which the matching was performed. The former could be exacerbated by the fact that CNV carrier individuals with the more extreme phenotypes are less likely to have 25 matched control subjects in the UKBB. To explore this hypothesis, we compared mean trait value or disease prevalence between the subset of CNV carrier individuals used for the matched-control analysis and the one excluded due to missing data or lack of a sufficient number of matched control subjects (Figure S6; Tables S5 and S6). Except for recurrent depressive disorder and forced vital capacity, all comparisons were non-significant (*p* ≥ 0.05), indicating that subsampling does not strongly impact our results. For recurrent depressive disorder, the only three duplication carrier individuals diagnosed with the disease were not included in the matched-control analysis (*p* = 0.03; Figure S6D), indicating that the non-significant effect of the duplication on recurrent depressive disorder (Figure 4D) is likely caused by subsampling. For forced vital capacity, excluded deletion carrier individuals exhibited a more pronounced phenotypic decrease than the ones retained for the matched-control analysis (*p* = 0.02; Figure S6R), suggesting that a more extreme difference would have been observed if these individuals had been included in the matched-control analysis (Figure 4R), likely making the association Bonferroni-significant. Conversely, the role of the CNV on anemia risk (Figure 4T) and cystatin C (Figure 4V) is likely driven by a combined effect of the CNV on adiposity and EA, and other factors on which matching was performed.

## Discussion

In this study, we perform a comprehensive PheWAS assessing the relation between 16p11.2 BP4-5 CNVs and 117 complex traits and diseases in the general population through four dosage mechanisms of action. Our results confirm the extreme pleiotropy of 16p11.2 BP4-5 rearrangements, with 46 traits associating with the CNV. In line with the more deleterious nature of the deletion, haploinsufficiency associated with 38 unique traits, while only 10 traits associated with the region’s duplication. Further emphasizing how the same genetic region can affect different traits through different dosage mechanisms, we identify traits for which the loss and gain of a copy had an opposite (e.g., BMI or platelet count) or, alternatively, a similar (e.g., hand grip strength or fluid intelligence score) consequences on the phenotype. Besides assessing the role of dosage in pleiotropy, we also estimated the fraction of associations that are likely to be secondary to some hallmark features of the CNV and validated through bidirectional MR that mediation is a likely scenario. While height did not mediate any associations, sixteen of the deletion-driven associations were found to be BMI dependent, thirteen of which received support from MR as being consequential to an initial increase in BMI. Conversely, the role of EA was more subtle, with only four associations showing confounding by that factor. Importantly, some associations were found to be independent of all the tested mediators, suggesting genuine direct pleiotropy of the region on musculoskeletal, hepatic, metabolic, neuropsychiatric, hematological, pulmonary, immune, and renal function.

Our findings have far-reaching consequences for clinical practice and highlight knowledge gaps. First, our results show that increased BMI in deletion carrier individuals drives numerous adult-onset comorbidities. Studies have shown that weight gain in 16p11.2 BP4-5 deletion carrier individuals starts during early childhood, to rapidly progress to obesity.^10^^,^^43^^,^^45^^,^^46^^,^^47^ This emphasizes the importance of following pediatric cases by a dedicated team of endocrinologists and nutritionists who can implement a weight control strategy at an early age to attenuate ensuing adult co-morbidities. Second, we show that some other traits are affected independently of the CNV’s effect on BMI and education. Besides recapitulating well-established hallmark features, such as the CNV’s negative impact on cognitive ability or the duplication-specific risk of bipolar affective disorder, we also link the CNV with milder afflictions of systems that had previously been implicated in clinical cohorts. For instance, increased risk for AKI might be the consequence of subclinical structural defects of the kidney that could affect renal function in the long term, paralleling the predisposition of deletion carrier individuals to congenital anomalies of the kidney and urinary tract.^48^^,^^49^^,^^50^ Similarly, increased risk for pneumonia might reflect an impaired immune system that is exacerbated into full-blown immunodeficiency in deletion carrier individuals that also present with a loss-of-function variant in *CORO1A*^51^ (MIM: 605000). Other traits that are affected through BMI-independent mechanisms, such as heel bone mineral density, platelet counts, pulmonary function, and liver enzymes, have not been linked with the CNV in clinical cohorts and future research should establish how often these traits are altered in carrier individuals and which are the molecular mechanisms that mediate this pleiotropy. These could be explored by gene-to-trait mapping strategies such as rare variant gene burden tests,^52^^,^^53^ as well as MR^54^ or colocalization^55^ that integrate association signals from common SNP-GWAS with transcriptomic and proteomic data to pinpoint genes linked with specific phenotypes. These data could also be leveraged to generate gene-by-trait association matrices whose clustering may reveal groups of traits with shared underlying genetic influences and for which CNV associations are more likely to disappear upon adjustment for one another. Thirdly, our results expose intriguing findings, casting light on questions that remain unanswered by the current study. For instance, the BMI-dependent association of the deletion with type 1 diabetes could be driven by misdiagnosing type 2 diabetes as type 1 due to early-onset diabetes following early-onset obesity. We also identify an association between the deletion and decreased serum creatinine levels. Serum creatinine levels are typically elevated in patients with renal dysfunction, as is the case for many deletion carrier individuals. We speculate that these results could be the consequences of reduced hepatic function and/or muscle mass, both of which are present among deletion carrier individuals. Similarly, it remains unclear whether elevated levels of alkaline phosphatase—for which levels of specific isoenzymes were not determined in UKBB—reflect hepatic, renal, or skeletal dysfunction. Validation of these hypotheses requires in-depth phenotypic characterization of carrier individuals’ medical records but will be crucial to better define the molecular pathophysiology of 16p11.2 BP4-5 CNV carrier individuals and hopefully lead to actionable insights related to the management of the condition’s co-morbidities.

Our study is not without limitations. First, by assessing a relatively homogeneous cohort, our study likely misses pleiotropic consequences that are expressed only in certain genetic or environmental backgrounds, a phenomenon exacerbated by the relatively small absolute number of CNV carrier individuals which hinders our statistical power. Future studies are needed to confirm trends that we observe at sub-significant level. Second, we decided to focus on only three covariates, which based on the literature, represent strong candidates to mediate indirect pleiotropic consequences of the region’s rearrangement. While height and BMI can be measured with relatively high accuracy, EA offers only a rough and imperfect proxy for cognitive function and socioeconomic status, possibly explaining its weaker mediatory role. Other factors that we did not assess might mediate the relation between 16p11.2 BP4-5 CNVs and some of the associated traits. In particular, we highlight the lack of good proxies for socioeconomic status that can be genetically instrumented and caution that BMI and height have also been shown to partly capture socioeconomic status.^56^ Third, the conducted MR analysis comes with its own limitations, namely violation of the exclusion-restriction assumption via correlated pleiotropy, which may have resulted in false positive mediator-to-trait causal effects.^57^^,^^58^ Indeed, Q heterogeneity statistics for IVW MR estimates were often significant, possibly indicating that some of the instruments are pleiotropic. Modern MR studies that use summary statistics based on hundreds of thousands of samples and rely on hundreds of instrumental variables—as is the case in our analyses—are increasingly powered to detect even minute amounts of pleiotropy, resulting in significant Q statistics. To mitigate this risk, we performed sensitivity analyses based on three additional MR methods that yielded globally consistent results. In any case, if both adjusted and unadjusted regression analyses show a significant CNV effect, we can convincingly suggest that independent pleiotropic mechanisms are at play. Finally, while our study brings us a step closer to understanding the pleiotropy of the region, it fails to provide molecular insights into mechanisms of pleiotropy, for which experimental approaches and leveraging of other mutational classes offer promising avenues.

In conclusion, our study provides a framework to start disentangling the complex pleiotropic patterns associated with genomic disorders. For 16p11.2 BP4-5, the latter appears to be a mixture of indirect effects mediated by the impact of the CNV on adiposity and cognition and direct effects on a broad range of physiological systems. This suggests that independent molecular mechanisms are involved in translating dosage changes into the many co-morbidities linked to the genomic disorder.

## Data and code availability

All data produced by this study are available in the supplemental tables. Code is available at https://github.com/cauwerx/16p11.2_BP4-5_pleiotropy.

## Acknowledgments

We thank UKBB biobank participants for sharing their data. Computations were performed on the Urblauna server from the University of Lausanne. The study was funded by the 10.13039/501100001711Swiss National Science Foundation (31003A_182632 to A.R., 310030_189147 to Z.K.), Horizon2020 Twinning projects (ePerMed 692145 to A.R.), and the Department of Computational Biology (Z.K.) and the Center for Integrative Genomics (A.R.) from the 10.13039/501100006390University of Lausanne.

## Author contributions

C.A. performed all analyses, except for MR analyses conducted by S.M.; Z.K. supervised statistical analyses; C.A., Z.K., and A.R. interpreted the data; C.A. generated the figures and drafted the manuscript; Z.K. and A.R. made critical revisions; and all authors approved the final manuscript.

## Declaration of interests

The authors declare no competing interests.

## Web resources

OMIM, https://www.omim.org/

## Supplemental information

Data S1. Tables S1–S6

Document S2. Article plus supplemental information
