# Supplementary material for: Disentangling mechanisms behind the pleiotropic effects of proximal 16p11.2 BP4-5 CNVs
Source: Am J Hum Genet. 2024 Sep 26;111(11):2347–61. doi: 10.1016/j.ajhg.2024.08.014 (PMC11568757; doi:10.1016/j.ajhg.2024.08.014)
Supplement: Document S1. Figures S1–S6 [file mmc1.pdf]

The American Journal of Human Genetics, Volume *111*

Supplemental information

Disentangling mechanisms behind the pleiotropic effects of proximal 16p11.2 BP4-5 CNVs

Chiara Auwerx, Samuel Moix, Zoltán Kutalik, and Alexandre Reymond

SUPPLEMENTAL FIGURES

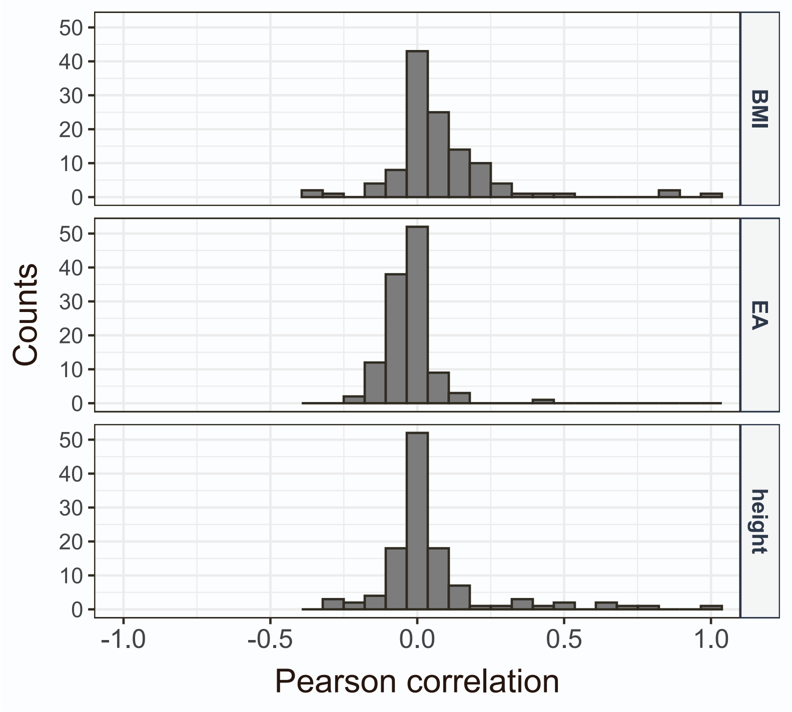

Figure S1. Correlation between phenotypes and putative mediators.
Histograms showing the distribution of Pearson correlation coefficients between the 117 phenotypes assessed for association with 16p11.2 BP4-5 CNVs through phenome-wide association studies and three putative mediatory traits: body mass index (BMI; top), educational attainment (EA; middle), and height (bottom).

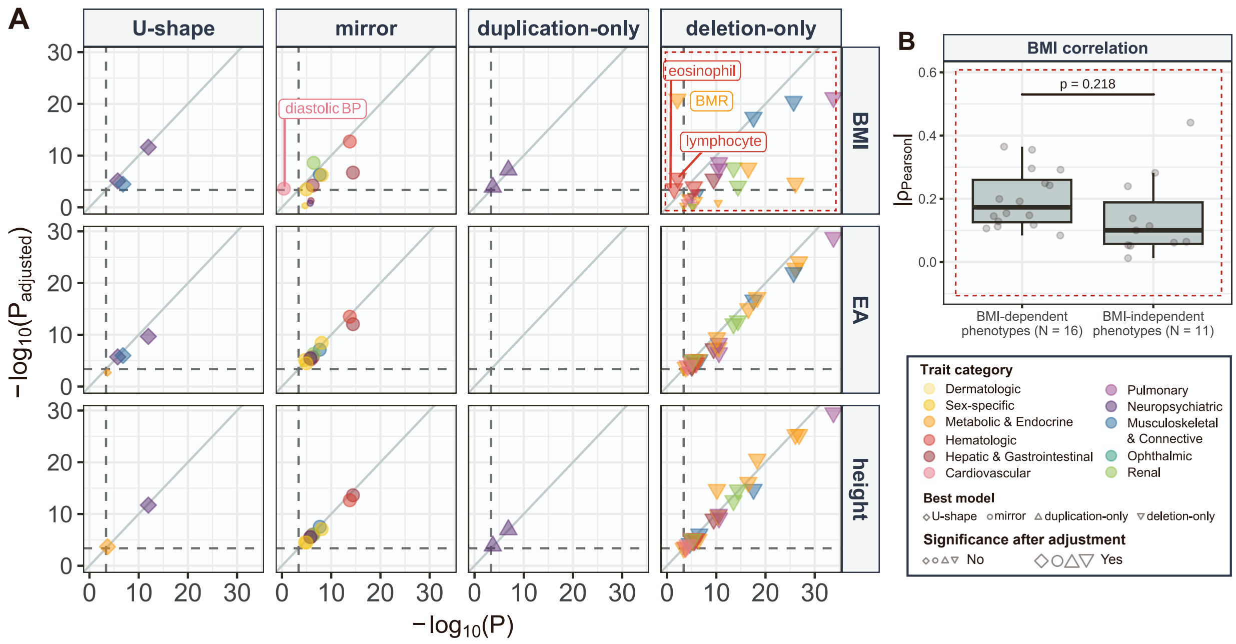

Figure S2. Adjustment for potential mediators of 16p11.2 BP4-5 pleiotropy.

(**A**) Negative logarithm of p-values of 16p11.2 BP4-5 CNV effect on traits with adjustment for potential mediators (y-axis) – i.e., body mass index (BMI; top row), educational attainment (EA; middle row), and height (bottom row) – against those without adjustment (x-axis), stratified according to the best (i.e., most significant) association model (shape; columns). Only associations that were significant prior to or become significant after adjustment are plotted. Traits are colored according to physiological systems. Size reflects whether the effect is Bonferroni significant ($p \leq 0.05/117 = 4.3 \times 10^{-4}$) after adjusting for the potential mediator (large) or not (small). Traits that become Bonferroni significant after adjustment for mediator are labeled. Grey diagonal represents the identity line; Dark grey dashed lines represent the Bonferroni threshold. (**B**) Absolute value of the Pearson coefficients of correlation of BMI with traits that are significantly associated with the deletion (red dashed square in (A)), stratified according to whether the association is lost ("BMI-dependent") or not ("BMI-independent") after adjustment for BMI. The P-value compares the two groups with a two-sided t-test. Number of traits is indicated as N. BMR = basal metabolic rate; diastolic BP = diastolic blood pressure; eosinophil = eosinophil count; lymphocyte = lymphocyte count.

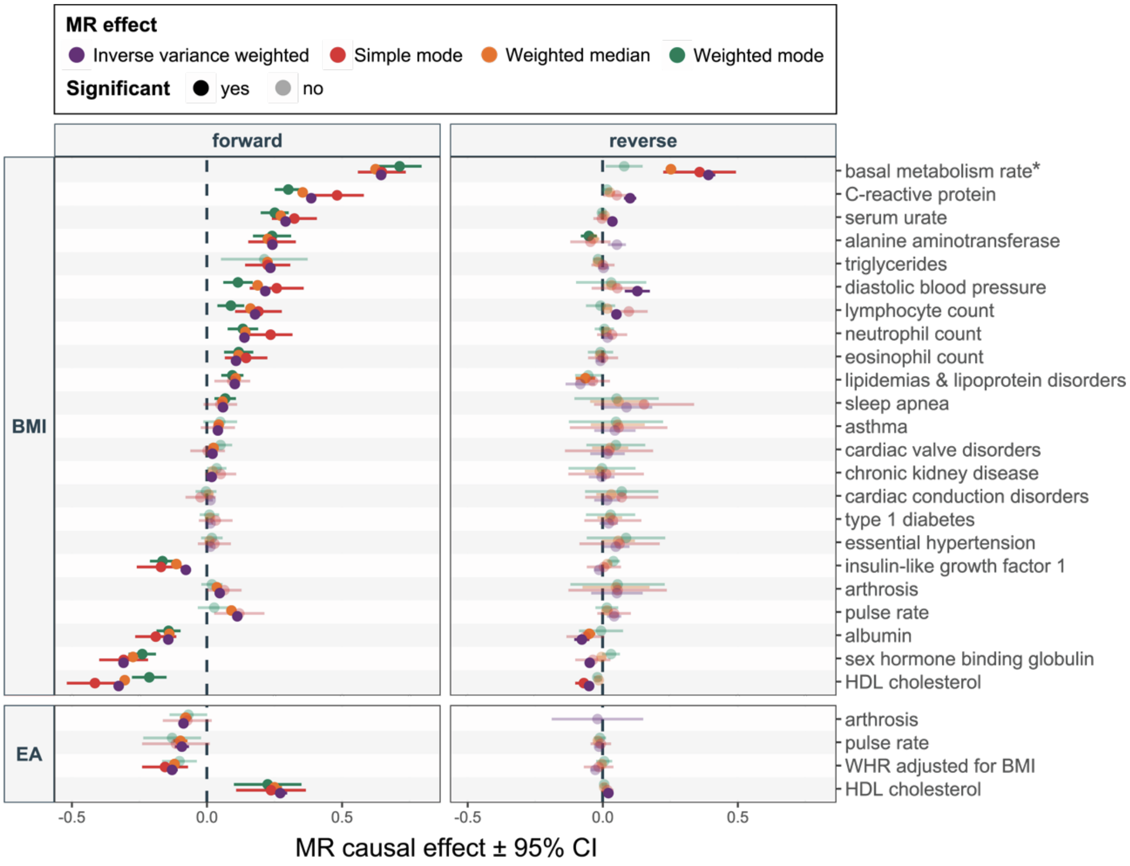

Figure S3. Mendelian randomization sensitivity analysis.

Sensitivity analysis of the Mendelian randomization (MR) results for the 27 mediator (left y-axis) to phenotype (right y-axis) pairs that were tested. Estimates with 95% confidence interval (CI) of the forward (mediator on phenotype; left column) and reverse (phenotype on mediator; right column) MR causal effects (x-axis), according to four different MR methods, are plotted. Semi-transparent effects do not survive Bonferroni correction ($p \leq 0.05/54 = 9.3 \times 10^{-4}$). Phenotypes labeled with "*" exhibit a Pearson correlation between 0.4-0.7 with the mediator. BMI = body mass index; EA = educational attainment; HDL = high-density lipoprotein; WHR = waist-to-hip ratio.

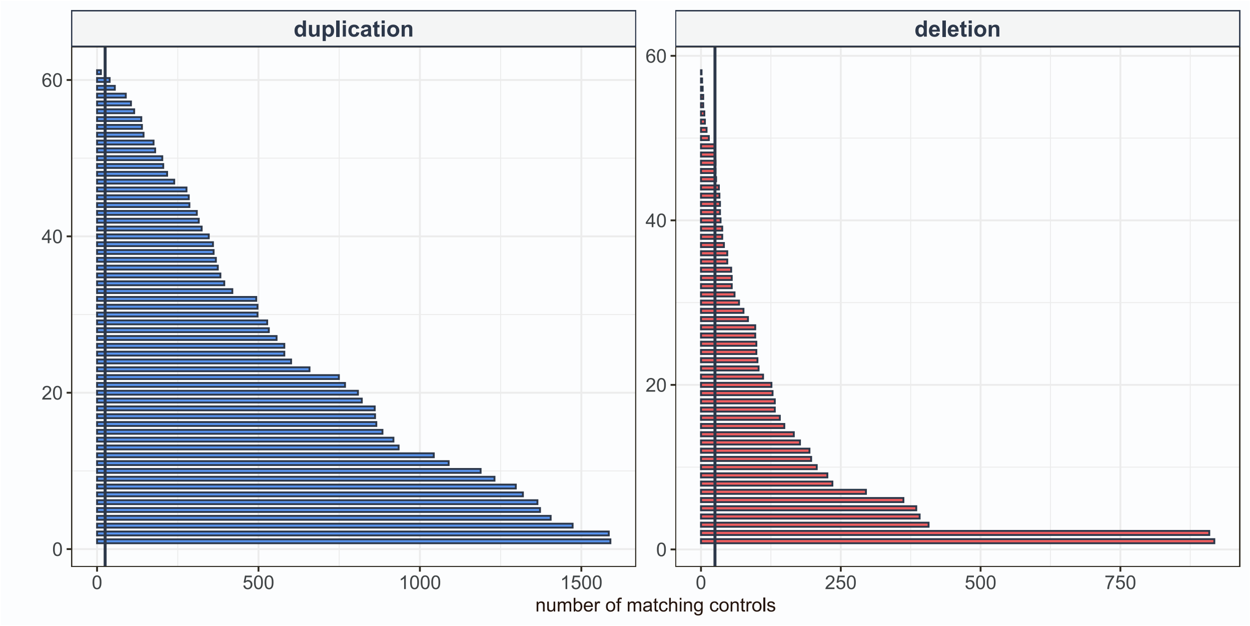

Figure S4. Number of matched controls per 16p11.2 BP4-5 CNV carrier.
Total number of identified matched controls (x-axis) per 16p11.2 BP4-5 duplication (N = 61; blue; left) and deletion (N = 58; red; right) carrier (y-axis). The black vertical line represents the cutoff of 25 randomly sampled matched controls per CNV carrier. In total 60 duplication and 49 deletion carriers passed this threshold and were retained for matched-control analysis.

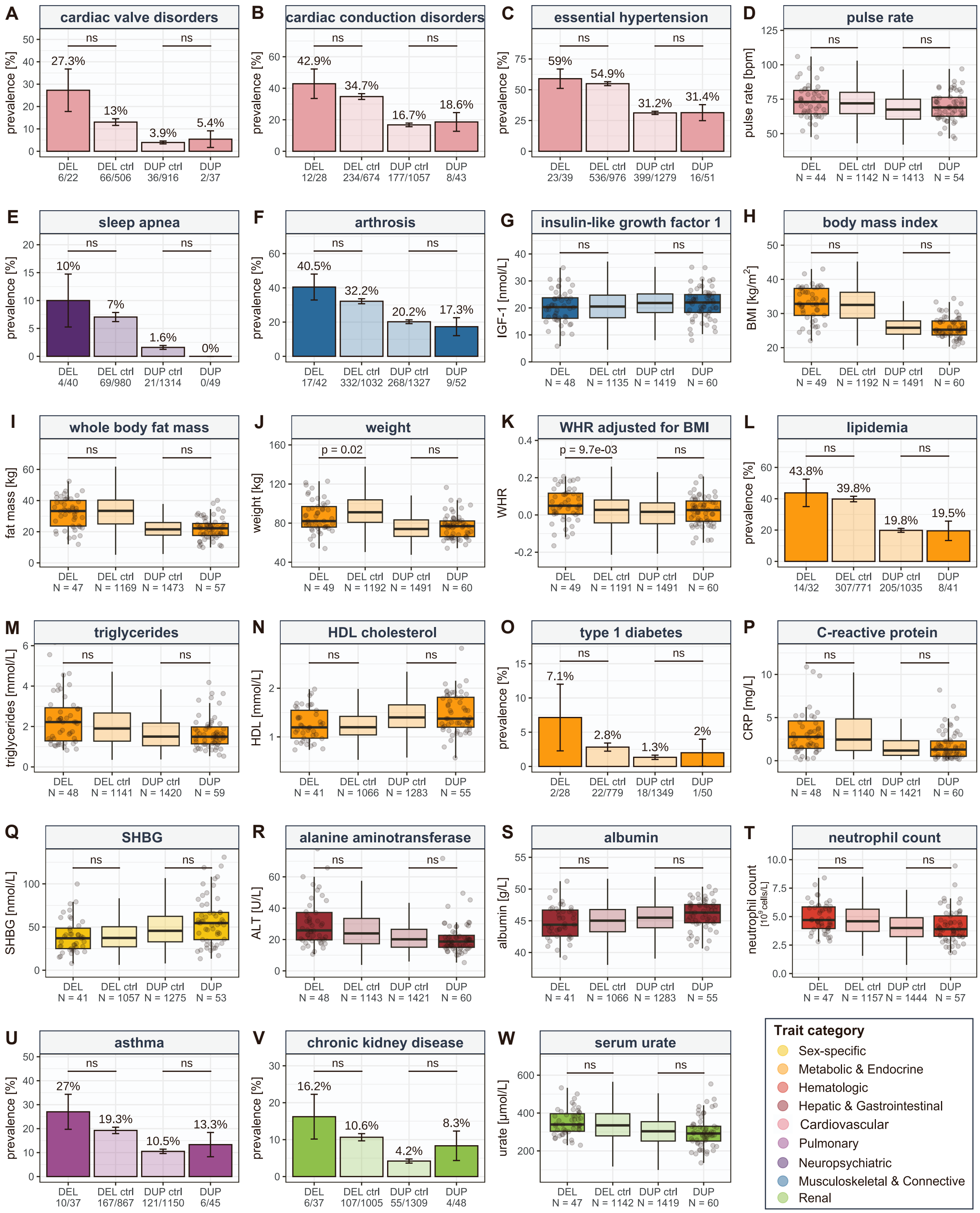

Figure S5. 16p11.2 BP4-5 CNV carriers matched-control analyses: negative control traits.
(**A**-**W**) Comparison between deletion (DEL) and duplication (DUP) carriers (dark shade) and their respective matched controls (DEL or DUP ctrl; lighter shade) for 23 traits that were significantly associated with 16p11.2 BP4-5 CNVs in our phenome-wide association study ($p \le 0.05/117 = 4.3 \times 10^{-4}$) but whose association was dependent on adjustment for mediators/had high trait-mediator correlation. For quantitative traits, data are represented as boxplots without outliers

and data points for CNV carriers are shown as grey dots. Sample size is indicated as N. P-values of a two-sided t-test comparing CNV carriers to matched controls are indicated. For diseases, prevalence in percentage with standard errors is depicted. Number of cases and total sample size are reported. P-values of two-sided Fisher tests comparing CNV carriers to matched controls are reported. “ns” indicates $p > 0.05$. Traits are colored according to physiological systems. ALT = alanine aminotransferase; BMI = body mass index; CRP = C-reactive protein; HDL = high-density lipoprotein; IGF-1 = insulin-like growth factor 1; lipidemia = lipidemias & lipoprotein disorders; SHBG = sex hormone binding globulin; WHR = waist-to-hip ratio.

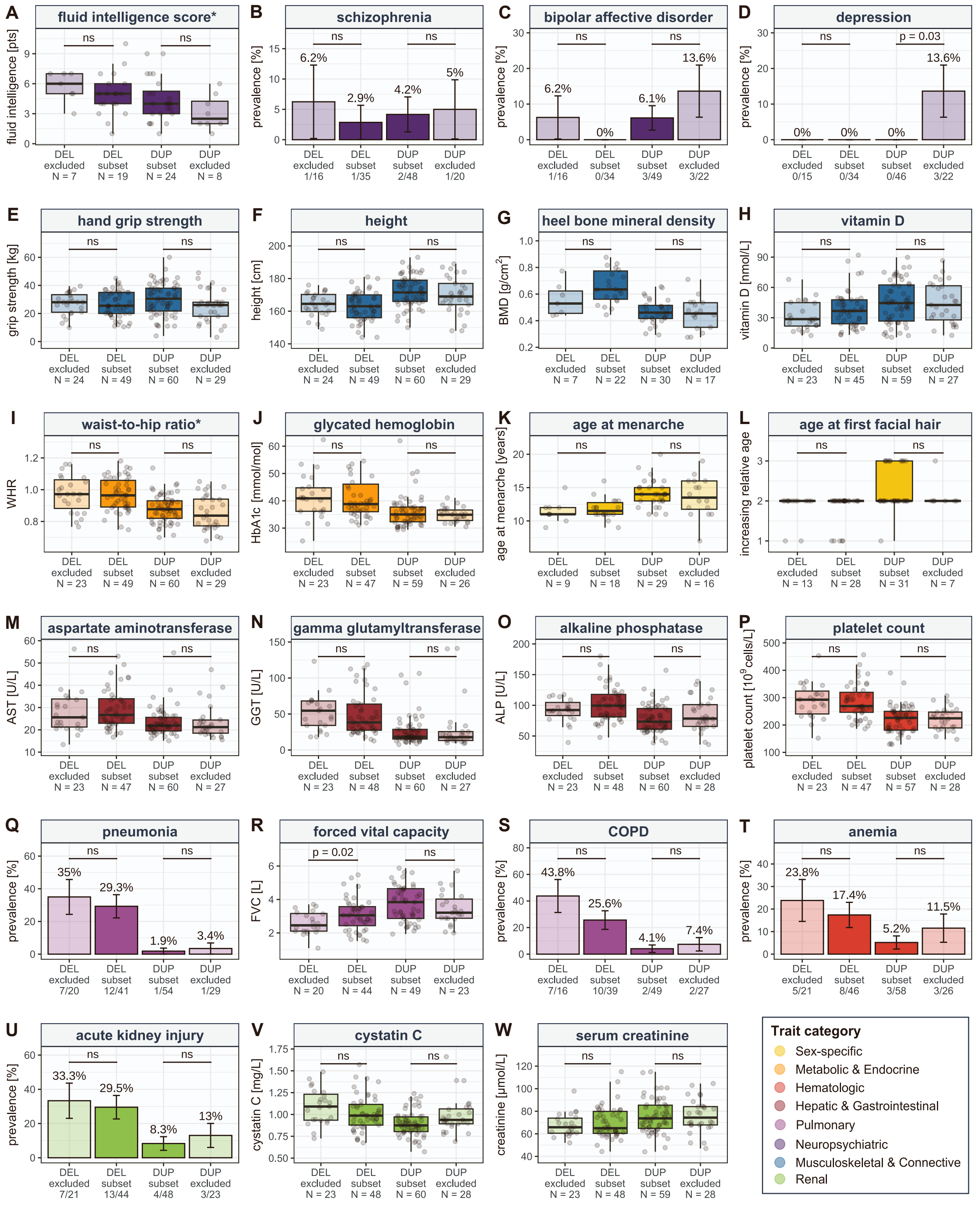

Figure S6. Impact of CNV carriers subsampling on matched-control analyses.
(**A**-**W**) Comparison between deletion (DEL; N_{max} = 49) or duplication (DUP; N_{max} = 60) carriers that were in the subset ("subset"; darker shade) used for the matched-control analyses against deletion (DEL; N_{max} = 24) or duplication (DUP; N_{max} = 29) carriers that were not included due to lack of data ("excluded"; lighter shade) for 23 traits which remained Bonferroni-significant ($p \leq 0.05/117 = 4.3 \times 10^{-4}$) after adjusting for body mass index (BMI), educational attainment (EA),

or height in Figure 3. Phenotypes labeled with “*” exhibit a Pearson correlation between 0.4-0.7 with either BMI or EA. For quantitative traits, data are represented as boxplots without outliers and data points for CNV carriers are shown as grey dots. Sample size is indicated as N. P-values of two-sided t-test comparing CNV carriers to matched controls are reported. For diseases, prevalence in percentage with standard errors is depicted. Number of cases and total sample size is indicated. P-values of two-sided Fisher tests comparing CNV carriers to matched controls are reported. “ns” indicates $p > 0.05$. Traits are colored according to physiological systems. ALP = alkaline phosphatase; AST = aspartate aminotransferase; BMD = bone mineral density; COPD = chronic obstructive pulmonary disease; depression = recurrent depressive disorder; FVC = forced vital capacity; GGT = gamma-glutamyltransferase; HbA1c = glycated hemoglobin; WHR = waist-to-hip ratio.
